# Hypoxia-Inducible Factor 1α (HIF1α) Suppresses Virus Replication in Human Cytomegalovirus Infection by Limiting Kynurenine Synthesis

**DOI:** 10.1128/mBio.02956-20

**Published:** 2021-03-23

**Authors:** Lisa M. Wise, Yuecheng Xi, John G. Purdy

**Affiliations:** a Department of Immunobiology, University of Arizona, Tucson, Arizona, USA; b BIO5 Institute, University of Arizona Tucson, Tucson, Arizona, USA; c Cancer Biology Interdisciplinary Program, University of Arizona, Tucson, Arizona, USA; University of North Carolina, Chapel Hill

**Keywords:** human cytomegalovirus, aryl hydrocarbon receptor, hypoxia-inducible factor 1α, indoleamine 2,3-dioxygenase 1, kynurenine, metabolomics

## Abstract

Human cytomegalovirus (HCMV) replication depends on the activities of several host regulators of metabolism. Hypoxia-inducible factor 1α (HIF1α) was previously proposed to support virus replication through its metabolic regulatory function. HIF1α protein levels rise in response to HCMV infection in nonhypoxic conditions, but its effect on HCMV replication was not investigated. We addressed the role of HIF1α in HCMV replication by generating primary human cells with HIF1α knocked out using CRISPR/Cas9. When HIF1α was absent, we found that HCMV replication was enhanced, showing that HIF1α suppresses viral replication. We used untargeted metabolomics to determine if HIF1α regulates metabolite concentrations in HCMV-infected cells. We discovered that in HCMV-infected cells, HIF1α suppresses intracellular and extracellular concentrations of kynurenine. HIF1α also suppressed the expression of indoleamine 2,3-dioxygenase 1 (IDO1), the rate-limiting enzyme in kynurenine synthesis. In addition to its role in tryptophan metabolism, kynurenine acts as a signaling messenger by activating aryl hydrocarbon receptor (AhR). Inhibiting AhR reduces HCMV replication, while activating AhR with an exogenous ligand increases virus replication. Moreover, we found that feeding kynurenine to cells promotes HCMV replication. Overall, our findings indicate that HIF1α reduces HCMV replication by regulating metabolism and metabolite signaling.

## Abstract (precis)

Viruses, including human cytomegalovirus (HCMV), reprogram cellular metabolism using host metabolic regulators to support virus replication. Alternatively, in response to infection, the host can use metabolism to limit virus replication.

## INTRODUCTION

Human cytomegalovirus (HCMV) is a herpesvirus that establishes lifelong asymptomatic infection in most people. HCMV infection in people with a compromised immune system causes disease that can lead to death. Additionally, HCMV infection is a leading cause of congenital disabilities (1). Replication of HCMV depends on evading cellular innate antiviral responses and hijacking host processes to support virus replication. Infection alters activity in many pathways in the host metabolic network, such as increasing glycolysis and the flow of carbons into lipid synthesis to support virus replication (2–8).

Metabolic reprogramming following HCMV infection involves hijacking the activity of host metabolic regulators (2). Hypoxia-inducible factor 1α (HIF1α) is a metabolic regulator that is altered by HCMV infection. Cell sensing of HCMV infection increases HIF1α protein levels under conditions with normal oxygen levels (i.e., normoxia) (9). Infected cells sustain HIF1α activity upon expression of HCMV early genes (10). These previous works proposed that HIF1α was induced by HCMV to support virus replication and pathogenesis (9, 10). However, the biological significance of HIF1α in HCMV infection remains unaddressed.

Here, we examined the role of HIF1α in HCMV replication by infecting CRISPR/Cas9 engineered HIF1α knockout (KO) primary human cells in normoxia. In contrast to the proposal in the previous work, we find that HCMV replication is enhanced in HIF1α KO cells. This observation suggests that HIF1α activity in infected cells alters metabolism as a protective strategy to limit viral infection. Some metabolites or metabolic activities support antiviral responses (11). We determined if HIF1α regulation of metabolism is necessary for limiting HCMV replication using untargeted metabolomics. We identified one of the metabolites regulated by HIF1α in HCMV-infected cells as kynurenine (KYN). Intracellular and extracellular KYN levels were markedly increased in HCMV-infected cells lacking HIF1α. KYN is a metabolite in tryptophan degradation that is made by the indoleamine 2,3-dioxygenase 1 (IDO1) pathway. We show that blocking IDO1 reduced HCMV replication, suggesting that KYN synthesis supports HCMV replication. In addition to its metabolic function, KYN also acts as a signaling messenger by activating the aryl hydrocarbon receptor (AhR). We find that blocking AhR signaling reduced HCMV replication, suggesting that HIF1α imparts an antiviral effect by decreasing KYN synthesis and AhR activation in HCMV infection. Overall, our findings demonstrate that HIF1α in HCMV-infected cells under normoxic conditions regulates metabolism in a way that reduces virus replication through a functional connection between HIF1α, IDO1, and AhR.

## RESULTS

### HIF1α reduces HCMV replication.

**FIG 1:**
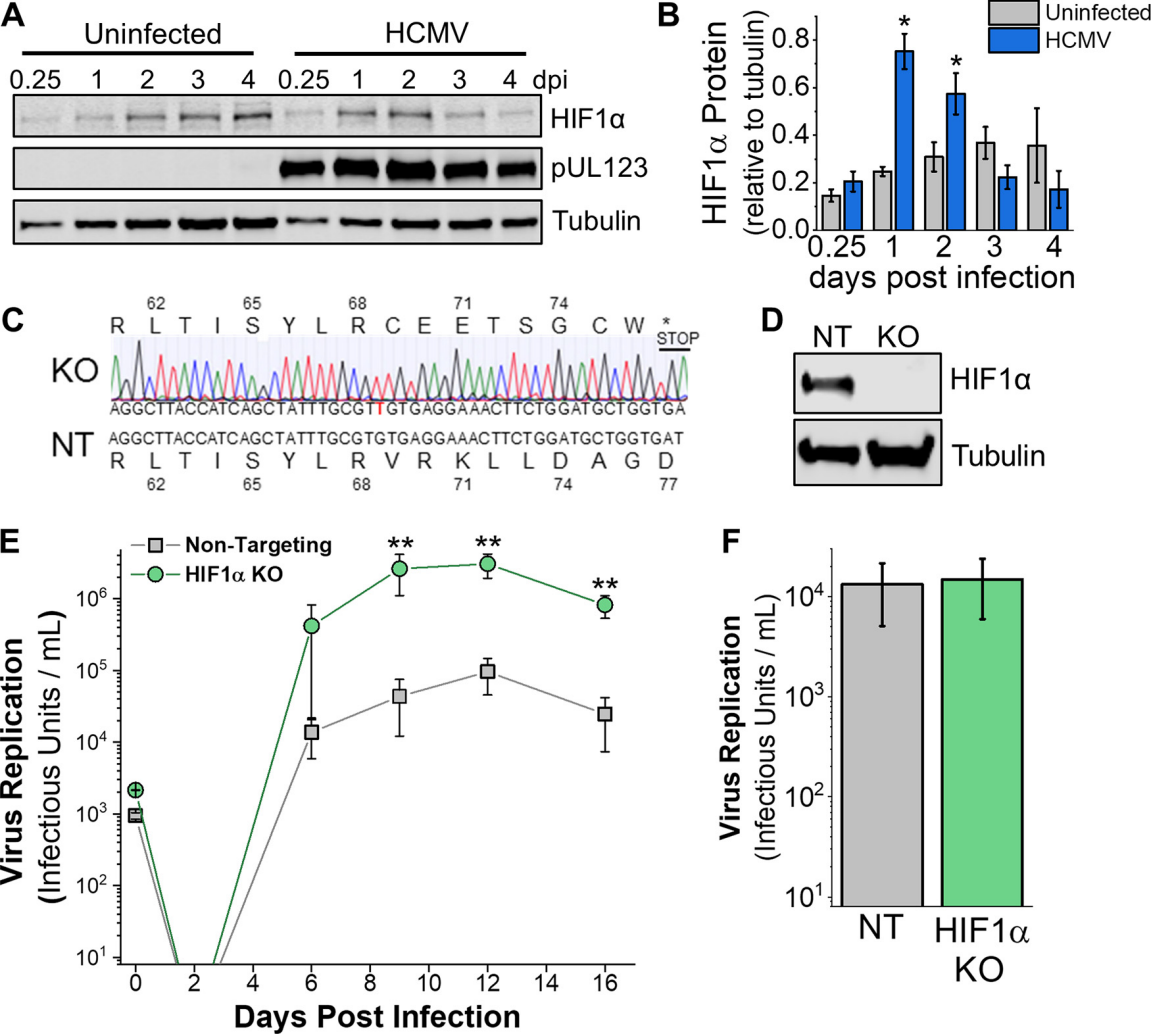
HIF1α is induced by HCMV infection and limits virus replication in normoxia. (A) HIF1α protein levels in uninfected and HCMV-infected fibroblasts cells were determined by Western blotting. Cells were infected at an MOI of 3 infectious units per cell with strain TB40/E when cells were 80% confluent. Uninfected cells continued to grow, reaching full confluence by day 1 to 2. (B) Quantification of HIF1α protein relative to tubulin in cells described for panel A. Data are means ± SD, and significance was determined by unpaired *t* test (*n* = 3) (*, *P* < 0.05). (C) HIF1α KO cells were generated using CRISPR/Cas9 in HFF-hTERT cells. Shown is the indel sequencing for HIF1α KO and the sequence from cells treated with a nontargeting (NT) gRNA. (D) HIF1α protein level in NT and HIF1α KO cells following 24 h treatment with 100 μM CoCl_2_ to mimic hypoxia. (E) HIF1α KO and NT cells were infected at an MOI of 0.05, and virus growth was measured by 50% tissue culture infective dose (TCID_50_) at the indicated days postinfection (dpi). Data are means ± SD, and significance was determined by ANOVA and Tukey’s test (*n* = 3) (**, *P* < 0.01) (day 6 excludes an outlier, which is shown in Fig. S1). (F) Virus replication in HIF1α KO and NT cells at an MOI of 3 (4 dpi; *n* = 3).

HCMV infection using the AD169 or Towne fibroblast-adapted strains increases HIF1α protein levels in the presence of oxygen (9). We expanded on this observation by determining if HIF1α protein levels are affected by infection with the TB40/E strain, which has not been extensively passaged in fibroblasts. Quantification of HIF1α protein levels, after normalization to tubulin protein levels, was done in cells infected with TB40/E at a multiplicity of infection (MOI) of 3 infectious units per cell. HIF1α protein levels showed 3-fold and ∼2-fold increases in TB40/E-infected cells relative to uninfected cells at 1 and 2 days postinfection (dpi), respectively (Fig. 1A and B). HIF1α protein levels were highest at 1 dpi in cells infected with TB40/E, similar to the lab-adapted strains (9, 10). In HCMV-infected cells, HIF1α levels rose and then decreased after 2 dpi (Fig. 1A). In contrast, HIF1α protein levels in uninfected cells rose throughout the 4-day time course (Fig. 1A). In our experiments, the cells were mock-infected or HCMV-infected when they were subconfluent and maintained under normoxic conditions throughout the time course. Under these conditions, the uninfected cells continued to grow until reaching full confluence. Since the HIF1α levels are normalized to tubulin and under normoxic growth conditions where HIF1α protein is typically degraded, these are unlikely to explain the observed increase in HIF1α in uninfected cells. A more likely possibility is that hypoxic microenvironments are created in the uninfected cell culture after reaching full confluence, which is known to increase HIF1α protein levels of cells in normoxia (12, 13). Next, we examined if HCMV infection alters HIF1α activity by measuring the expression of the gene encoding vascular endothelial growth factor (VEGF), which is transcriptionally regulated by HIF1α. VEGF transcripts were 20-fold higher in HCMV-infected cells than mock-infected cells, indicating that HIF1α is active in HCMV-infected cells maintained under normal oxygen conditions (see Fig. S1A in the supplemental material).

10.1128/mBio.02956-20.2 FIG S1 HCMV replication in NT and HIF1α KO cells. (A) VEGF transcripts were quantified at 2 dpi using reverse transcriptase quantitative PCR (RT-qPCR) relative to the housekeeping gene H6PD. Fibroblasts were mock infected or HCMV infected at an MOI of 3 in normoxia, as described for Fig. 1A and B. VEGF transcript levels are presented as means ± SD relative to mock-infected cells. Significance was determined using an unpaired *t* test, *n* = 3 (*, *P* < 0.05). (B) Shown are the individual growth curves for the three independent experiments described for Fig. 1E. At every time point, two to four independent TCID_50_ assays were performed for each sample. The error bars represent the SD of the two TCID_50_ assays. Download FIG S1, TIF file, 0.5 MB. Copyright © 2021 Wise et al. 2021 Wise et al. https://creativecommons.org/licenses/by/4.0/ This content is distributed under the terms of the Creative Commons Attribution 4.0 International license.

We generated HIF1α knockouts (KO) in primary human fibroblasts using CRISPR/Cas9 to determine the effect of HIF1α on HCMV replication. We identified HIF1α KO by sequencing (Fig. 1C). Our HIF1α KO clone contained a single nucleotide insertion that caused a frameshift that resulted in a premature stop codon. We confirmed the loss of HIF1α protein by Western blotting. We treated cells with cobalt chloride (CoCl_2_) to chemically mimic hypoxia and increase HIF1α protein levels. Consistent with our sequencing results, we observed no HIF1α protein in HIF1α KO cells (Fig. 1D). As a control, we generated CRISPR/Cas9 cells expressing a nontargeting (NT) guide RNA (gRNA) that does not recognize any human or HCMV gene. Next, we examined the ability of HCMV to replicate in HIF1α KO and NT cells. We infected HIF1α KO and NT control cells at a low MOI of 0.05 infectious unit per cell and quantified the production of new virus progeny over 16 days. At 9 dpi, HIF1α KO cells produced ∼60-fold more HCMV progeny than NT cells (Fig. 1E; Fig. S1B). At 12 and 16 dpi, ∼30-fold more infectious virus was produced by HIF1α KO cells than NT cells. Since the 16-day virus growth curves shown in Fig. 1E measure multiple rounds of HCMV replication, we next tested virus replication in a single replication cycle by infecting cells at an MOI of 3 and measuring infectious viral progeny at 4 dpi. Under these conditions, HCMV replication in HIF1α KO cells is similar to that in NT cells (Fig. 1F). Overall, our observations indicate that HIF1α suppresses HCMV replication at a low MOI and may contribute to an innate cellular antiviral response that is suppressed or not measured in a single-cycle high-MOI virus replication assay.

### Metabolic regulation by HIF1α in HCMV-infected cells.

Since HIF1α regulates metabolism (14–16), we investigated if HIF1α controls a metabolic barrier to HCMV replication. We used an untargeted liquid chromatography high-resolution tandem mass spectrometry (LC-MS/MS) metabolomic approach to determine if HIF1α regulates metabolism in HCMV-infected cells. In these experiments, we measured intracellular and extracellular metabolites extracted from infected HIF1α KO and NT cells under normoxic conditions at 2 dpi. We selected 2 dpi because HIF1α protein levels are elevated at 1 to 2 dpi (Fig. 1A), HIF1α activity was high at 2 dpi, as measured by VEGF transcript levels (Fig. S1A), and significant metabolic reprogramming by the virus occurs at 2 dpi (3, 4, 17). First, we analyzed the untargeted data set against a library of metabolites from several metabolic pathways, including glycolysis, tricarboxylic acid (TCA) cycle, amino acids, and nucleotide metabolism. The library was built using commercially purchased metabolite standards that we used to define LC retention times and MS/MS spectral features using our LC-MS/MS methods. These defined retention times and MS/MS features were used to match peaks in the untargeted data to provide a list of defined (i.e., known) metabolites. Of the approximately 50 metabolites examined using this method, the abundance of most were unaltered in the HCMV-infected HIF1α KO cells compared to infected NT control cells (Fig. S2A and B). Of the eight metabolites altered by HIF1α KO, four are involved in nucleotide metabolism and three are intermediates in glycolysis or TCA cycle.

**FIG 2:**
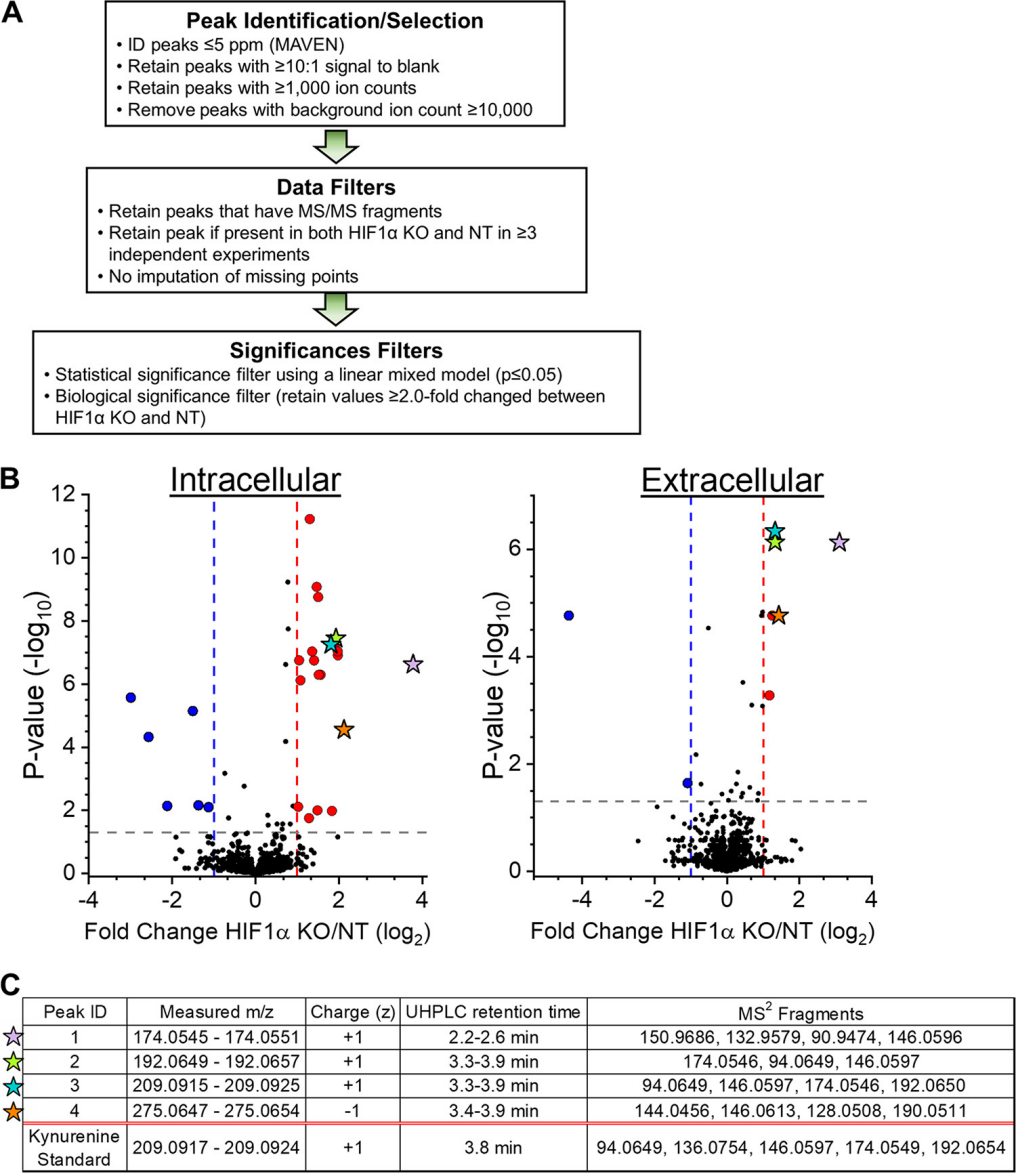
Untargeted metabolomics of HCMV-infected HIF1α-KO and NT control cells. (A) Schematic of the workflow for analyzing untargeted metabolomics LC-MS/MS data. Mass spectral peaks were selected and quantified using MAVEN software. Peaks were defined using a high mass accuracy setting of less than 5 ppm. Peaks were then selected if they had ≥1,000 ions to ensure the quality of quantitation above the limit of detection and the presence of MS/MS fragments that can be used to confirm metabolite identification. Finally, statistical significance was determined using linear mixed-effects models as described in Materials and Methods. A ≥2-fold change between HIF1α KO and NT was used to highlight potential biological significance. (B) At 2 dpi, intracellular and extracellular metabolites were extracted from HIF1α KO and NT cells infected with TB40/E at an MOI of 3. Metabolite peaks that passed the selection and data filters described in panel A are presented. Metabolites that decreased (blue) or increased (red) by ≥2-fold in HIF1α KO cells relative to NT cells are shown as colored circles or stars. Stars represent four metabolites that were increased in both the intracellular and extracellular environments of HIF1α KO relative to NT cells. The data are from 3 to 6 independent experiments. (C) Mass spectral and HPLC information for the four metabolites represented by stars in panel B. Also shown is information obtained using a commercially produced kynurenine standard analyzed using LC-MS/MS conditions used for untargeted metabolomics.

10.1128/mBio.02956-20.3 FIG S2 Metabolomic analysis of NT and HIF1α KO cells. (A) Metabolites in HCMV-infected NT and HIF1α KO cells were measured at 2 dpi. Metabolites were identified and quantified using LC-MS/MS. The data are fold change of HCMV-infected NT relative to uninfected NT cells and HCMV-infected HIF1α KO relative to uninfected HIF1α KO cells. The conditions are described in Fig. 2. (B) The data from panel A are presented as means ± standard errors of the means (SEM). Download FIG S2, TIF file, 0.7 MB. Copyright © 2021 Wise et al. 2021 Wise et al. https://creativecommons.org/licenses/by/4.0/ This content is distributed under the terms of the Creative Commons Attribution 4.0 International license.

Next, we used the metabolomic analysis software MAVEN to select mass spectral peaks unbiasedly (Table S1). MS1 peaks were selected using a high-resolution setting of ≤5 ppm. We filtered the data to determine peaks of significant interest for identification (Fig. 2A). In these filtering steps, we removed peaks caused by contaminants (i.e., background noise). We determined contaminants using two methods. First, potential contaminants from the buffers used in LC, the HPLC columns, and mass spectrometer were defined. Second, we identified potential contaminants from our extraction method. In each metabolomic experiment, cells were seeded in a six-well plate where at least one well contained no cells. The no-cell control was handled in parallel with the cells throughout the extraction and LC-MS/MS processes. Using these methods to identify contaminants, we retained MS peaks for further analysis only if they had a ≥10:1 signal-to-background ratio. Since MS/MS information is needed to confirm metabolite identification, the data were further filtered to retain MS1 peaks that had associated MS/MS fragments. Next, peaks were retained if they were observed in both HIF1α KO and NT samples in ≥3 independent experiments. The peaks that passed our filtering process were used to determine statistical significance between HIF1α KO and NT samples and organized by fold change in HIF1α KO relative to NT (Fig. 2B).

10.1128/mBio.02956-20.1 TABLE S1 Peak list from the untargeted metabolomic study described in Fig. 2. Download Table S1, XLSX file, 9.4 MB. Copyright © 2021 Wise et al. 2021 Wise et al. https://creativecommons.org/licenses/by/4.0/ This content is distributed under the terms of the Creative Commons Attribution 4.0 International license.

The abundances of most intracellular metabolites were similar in HIF1α KO and NT cells (Fig. 2B). We observed a ≥2-fold increase in 20 peaks and a ≥2-fold decrease in 6 peaks in HIF1α KO cells relative to NT cells. Fewer extracellular metabolites—including fewer metabolites that were HIF1α-dependent—were observed than in the intracellular fraction (Fig. 2B). Only two peaks in the extracellular environment were found to be significantly decreased in HIF1α KO cells relative to NT cells. These two peaks were different from the peaks significantly changed in the intracellular fraction by the loss of HIF1α.

In the extracellular fraction, we observed six peaks increased by HIF1α KO (Fig. 2B). Notably, four of these peaks were increased in both the intracellular and extracellular fractions by ≥2.5-fold in HIF1α KO cells relative to NT control cells (Fig. 2B, stars). We prioritized the identification of these four peaks. Peaks 2 and 3 had matching retention times of 3.3 to 3.9 min under reverse-phase LC conditions and shared several MS/MS fragments (i.e., 94.06, 146.05, and 174.05) (Fig. 2C). The data for these peaks were collected over a 9-month period that involved several batches of LC buffers and columns that resulted in a 0.6-min shift in retention time. The MS1 *m/z* of peak 3—209.09—matches the calculated *m/z* of kynurenine (KYN) within our defined 5-ppm range. We used a commercially obtained KYN standard to confirm its MS1 *m/z* and determine its retention time using our reverse-phase LC method and its MS/MS fragments using our metabolomic methods. The retention time and MS1 *m/z* for the KYN standard matched those we observed for peak 3 (Fig. 2C). Next, we examined the MS/MS fragments. The four MS/MS fragments of peak 3 matched four of the top five fragments we observed using the KYN standard (Fig. 2C), confirming the identification of peak 3 as KYN.

When defining the MS1 *m/z* of KYN in positive mode using the commercial KYN standard, we observed that electrospray ionization fragmented KYN to form an ion at 192.06 *m/z*. This ionization fragment matched the measured MS1 *m/z* for peak 2 within 5 ppm. The three MS/MS fragments observed for peak 2 matched three fragments observed using the KYN standard and fragments observed in peak 3 (Fig. 2C), providing evidence that both peaks represent KYN. We conclude that peaks 2 and 3 are KYN. Peaks 1 and 4 remain unidentified. Since the levels of KYN in human serum were previously found to be associated with HCMV infection (18, 19), we decided to focus on the role of KYN in virus replication.

### HCMV infection raises KYN levels, which are suppressed by HIF1α.

**FIG 3:**
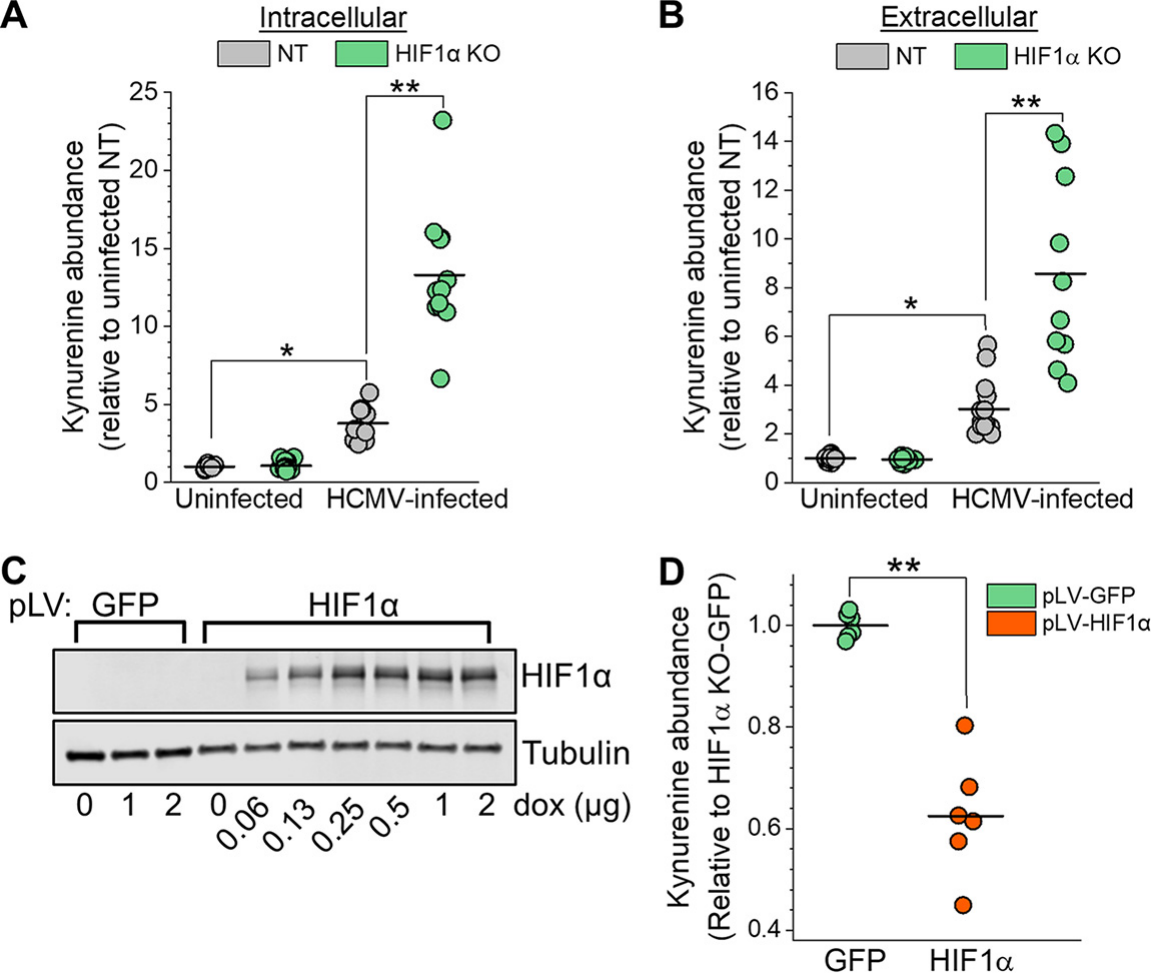
HIF1α suppresses kynurenine (KYN) levels in HCMV-infected cells. (A) Intracellular KYN levels in uninfected and HCMV-infected NT and HIF1α KO cells were measured by LC-MS/MS. Cells were infected at an MOI of 3, and metabolites were extracted at 2 dpi. The mean is represented by a black bar, and significance was determined by 2-way ANOVA and Tukey’s test (*n* ≥ 6) (*, *P* < 0.05; **, *P* < 0.01). (B) KYN levels in the extracellular environment under conditions described for part A. The mean is represented by a black bar, and significance was determined by 2-way ANOVA and Tukey’s test, *n* ≥ 6 (*, *P* < 0.05; **, *P* < 0.01). (C) Western blot analysis of HIF1α KO cells expressing GFP or HIF1α containing a silent mutation to mutate the Cas9 PAM recognition site. GFP and HIF1α expression was induced using a doxycycline (dox)-inducible system. Cells were treated with increasing concentrations of doxycycline for 2 days. (D) Intracellular KYN levels in HCMV-infected HIF1α KO cells expressing GFP or HIF1α-Cas9 PAM mutant. Cells were infected for 1 h at an MOI of 2 and then treated with 1 μg/ml doxycycline prior to extraction of metabolites at 2 dpi. The mean is represented by a black bar, and significance was determined using an unpaired *t* test (*n* = 3) (**, *P* < 0.01).

We further investigated the relationship between HIF1α and KYN levels. In uninfected cells growing in normoxia, the loss of HIF1α has little or no effect on intracellular or extracellular KYN levels (Fig. 3A and B). In the NT cells that express HIF1α, HCMV-infected cells have a 3.5-fold increase in intracellular KYN relative to uninfected cells at 2 dpi (Fig. 3A). Similarly, KYN levels were greater in the extracellular fraction of HCMV-infected NT cells than uninfected NT cells (Fig. 3B). These data demonstrate that HCMV infection, in cells with HIF1α, enhances intracellular and extracellular KYN levels.

Relative to HCMV-infected NT cells, infected HIF1α KO cells had 3.5-fold- and 2.5-fold-higher intracellular and extracellular KYN levels (Fig. 3A and B). Next, we sought to confirm that KYN is regulated by a HIF1α-dependent mechanism using a second independently engineered CRISPR/Cas9 HIF1α KO clone that contained a different gRNA. We generated a second HIF1α KO clone with two deletions that introduced frameshift mutations and failed to express HIF1α protein (Fig. S3A and B). At 2 dpi, KYN levels in HIF1α KO clone 1 and clone 2 were ≥2-fold higher than in NT cells, further supporting the conclusion that KYN levels are regulated by HIF1α (Fig. S3C).

10.1128/mBio.02956-20.4 FIG S3 Characterization of two HIF1α KO clones. (A) Description of CRISPR/Cas9 gRNAs used in this study. Two independent HIF1α KO clones were generated and confirmed by indel sequencing. The mutations identified and their consequences on HIF1α protein are shown. (B) HIF1α protein levels in NT and two HIF1α KO clones following 24 h treatment with 100 μM CoCl_2_ to mimic hypoxia. (C) KYN levels in HCMV-infected NT and HIF1α KO clones 1 and 2. Cells were infected at an MOI of 2 with TB40/E. At 2 dpi, metabolites were extracted and KYN was measured by LC-MS/MS. The means are represented by black bars. Four independent experiments were performed, each with duplicate samples per condition. Each of the 8 samples measured is represented by a dot. The data are fold change relative to HCMV-infected NT cells, and significance was determined by two-way ANOVA and Tukey’s test (*n* = 4) (**, *P* < 0.01). Download FIG S3, TIF file, 0.4 MB. Copyright © 2021 Wise et al. 2021 Wise et al. https://creativecommons.org/licenses/by/4.0/ This content is distributed under the terms of the Creative Commons Attribution 4.0 International license.

For further confirmation that HIF1α suppresses KYN levels in HCMV-infected cells, we re-expressed HIF1α in our KO cells using a doxycycline-inducible system (Fig. 3C). In these cells, we engineered the re-expressed HIF1α to contain a silent mutation that removes the Cas9 protospacer-adjacent motif (PAM) recognition site while leaving the amino acid sequence of the protein unaffected. As a control, we expressed green fluorescent protein (GFP) in HIF1α KO cells. We infected these cells for 1 h, washed them, and treated them with doxycycline to induce HIF1α or GFP expression. KO cells re-expressing HIF1α had almost 2-fold-lower levels of KYN than HIF1α KO cells expressing GFP (Fig. 3D). These observations provide further evidence that HIF1α suppresses KYN levels in HCMV-infected cells.

### IDO1 and AhR activities promote HCMV replication.

**FIG 4:**
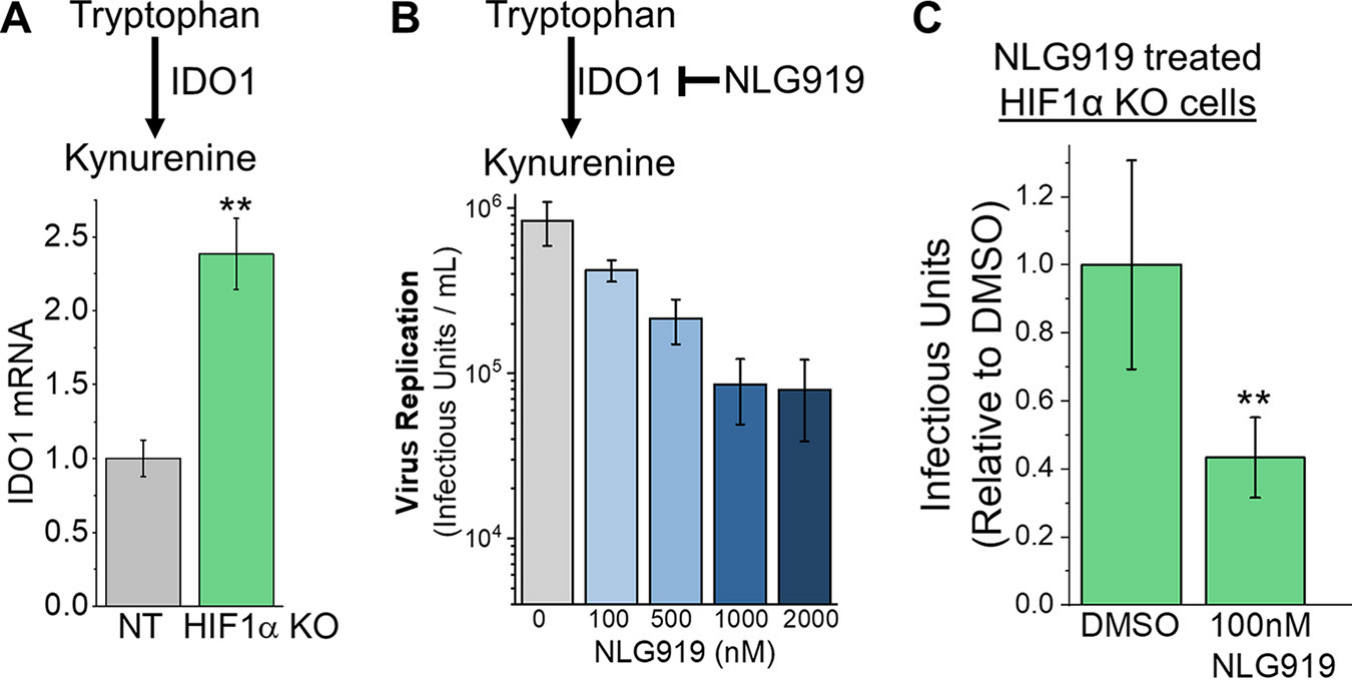
HIF1α suppresses IDO1, which supports HCMV replication. (A) IDO1 is the rate-limiting enzyme in KYN synthesis. IDO1 transcripts in HCMV-infected HIF1α KO and NT cells were measured at 2 dpi using RT-qPCR. Cells were infected at an MOI of 3 with TB40/E. The data are mean ± SD relative to IDO1 levels in HCMV-infected NT cells. Statistical significance was determined using an unpaired *t* test (*n* = 3) (**, *P* < 0.01). (B) NLG919 inhibits IDO1 activity. HCMV replication in non-genetically modified fibroblasts was measured following treatment with NLG919. HFFs were infected at an MOI of 1, and production of infectious progeny virus was measured at 5 dpi. At 2 dpi, the growth medium was replaced to replenish NLG919. The data are means ± SD (*n* = 3). (C) HCMV replication in HIF1α KO cells treated with 100 nM NLG919 was determined. HIF1α KO cells were infected at an MOI of 0.05, and infectious progeny virus was determined at 9 dpi. At 3 and 6 dpi, the growth medium was replaced to replenish NLG919. The data are means ± SD relative to DMSO, and significance was determined by unpaired *t* test (*n* = 3) (**, *P* < 0.01).

Since KYN is elevated in HCMV-infected HIF1α KO cells, we determined if the expression of the rate-limiting enzyme in KYN synthesis, indoleamine 2,3-dioxygenase 1 (IDO1), is regulated by a HIF1α-dependent mechanism. At 2 dpi, IDO1 transcripts were increased by >2-fold in HCMV-infected HIF1α KO cells relative to infected NT cells (Fig. 4A). Our observations that IDO1 transcripts, KYN levels, and HCMV replication are enhanced in HIF1α KO cells led us to hypothesize that IDO1 activity promotes HCMV replication. We tested this hypothesis by inhibiting IDO1 activity using the IDO1 inhibitor NLG919. First, we examined the effect of NLG919 treatment on HCMV replication in fibroblast cells that had not been genetically modified by CRISPR/Cas9 or any other means. Cells were infected at an MOI of 1 and then treated with NLG919 at concentrations ranging from 100 to 2,000 nM. Dimethyl sulfoxide (DMSO)-treated cells were used as a control. The medium was replaced at 2 dpi to renew the level of NLG919. At these conditions, NLG919 treatment had little or no effect on the survival of uninfected cells (Fig. S4A). At 5 dpi, the amount of infectious viral progeny produced was measured by determining the 50% tissue culture infective dose (TCID_50_). At 2,000 nM, NLG919 treatment reduced HCMV replication by 10-fold, relative to DMSO-treated control cells (Fig. 4B). NLG919 treatment at 100 to 1,000 nM reduced HCMV replication by 2- to 9-fold.

**FIG 5:**
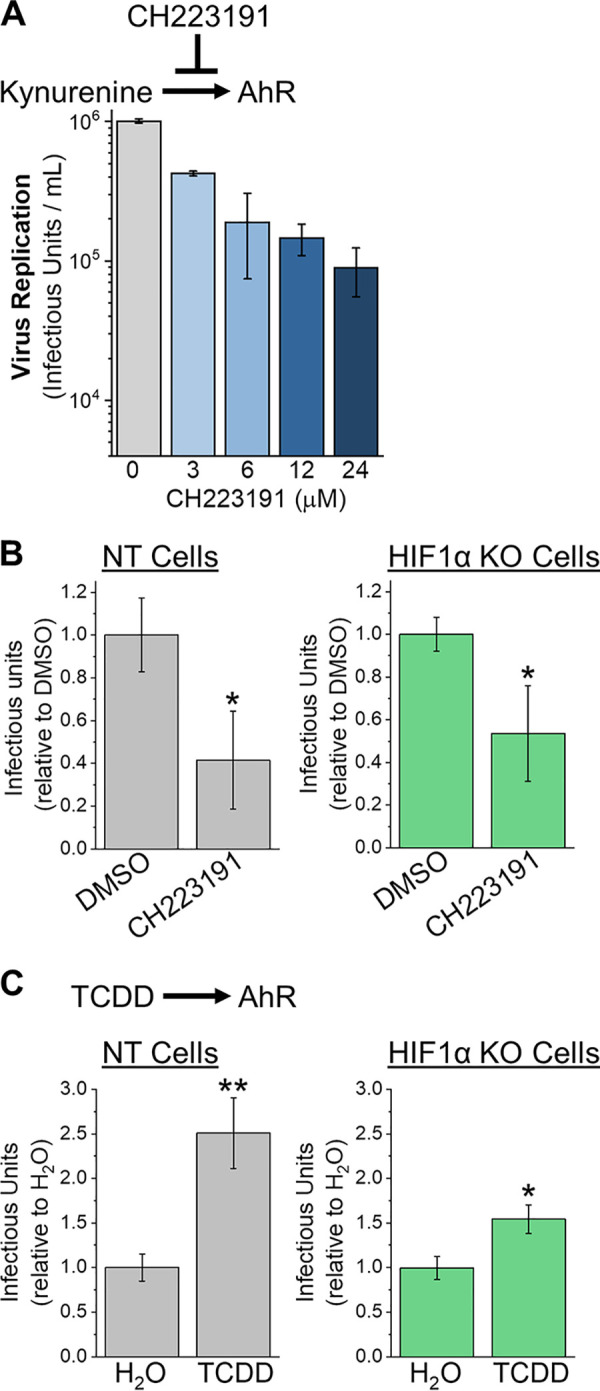
KYN receptor aryl hydrocarbon receptor (AhR) promotes HCMV replication. (A) CH223191 inhibits KYN activation of AhR. HCMV replication in primary human fibroblasts was measured following treatment with CH223191. HFFs were infected at an MOI of 1, and production of infectious progeny virus was measured at 5 dpi. At 2 dpi, the growth medium was replaced to replenish CH223191. The data are means ± SD (*n* = 3). (B) HCMV replication was measured in NT and HIF1α KO cells treated with 3 μM CH223191. Cells were infected at an MOI of 0.05, and infectious HCMV progeny was measured at 9 dpi. At 3 and 6 dpi, the growth medium was replaced to replenish CH223191. The data are means ± SD relative to DMSO treatment. Significance was determined using an unpaired *t* test (*n* = 3) (*, *P* < 0.05). (C) HCMV replication in cells treated with 3.1 nM TCDD, an exogenous AhR activator. Cells were infected at an MOI of 0.05, and infectious HCMV progeny was measured at 9 dpi. At 3 and 6 dpi, the growth medium was replaced to replenish TCDD. Since TCDD is liquid at room temperature and was directly diluted in the growth medium, tissue culture-grade water was used as a control. The data are means ± SD relative to water treatment. Significance was determined using an unpaired *t* test (*n* = 3) (*, *P* < 0.05; **, *P* < 0.01).

10.1128/mBio.02956-20.5 FIG S4 Survival of cells treated with NLG919, CH223191, and TCDD. (A) Percentage of uninfected HFF fibroblasts surviving treatment with 100 to 2,000 nM NLG919, 3 to 24 μM CH223191, or DMSO as a control for 5 days. These were the conditions used for Fig. 4B and 5A. Cell survival was measured using a LDH cytotoxicity kit (*n* = 3). Data are means ± SEM. (B) Percent survival of uninfected NT and HIF1α KO cells treated with 100 nM NLG919, 3 μM CH223191, or DMSO was determined. Similarly, cells were treated with 3.1 nM TCDD or water as a control. These conditions were used for Fig. 4C and 5B and C. Cell survival was measured using a LDH cytotoxicity kit (*n* = 4). Data are means ± SEM. Download FIG S4, TIF file, 0.3 MB. Copyright © 2021 Wise et al. 2021 Wise et al. https://creativecommons.org/licenses/by/4.0/ This content is distributed under the terms of the Creative Commons Attribution 4.0 International license.

We further tested if HCMV replication depends on IDO1 activity using HIF1α KO cells and a low MOI under the conditions used for the virus growth assays shown in Fig. 1E. In this case, the cells were treated at 1 h postinfection (hpi), and the medium was replaced every third day to renew the level of NLG919. At 9 dpi, the amount of infectious viral progeny produced was measured by TCID_50_. Since the cells were treated for 9 days, we focused on the lowest NLG919 concentration examined—100 nM—to limit any off-target effects treatment might have on the health of the cells. Under these conditions, 100 nM had little or no effect on cell survival (Fig. S4B). At 9 dpi, infectious HCMV progeny production was >2-fold lower in cells treated with 100 nM NLG919 than DMSO-treated cells (Fig. 4C). We conclude that IDO1 activity promotes HCMV replication in non-genetically modified primary human fibroblasts and in HIF1α KO cells.

IDO1 controls the synthesis of KYN, which is an important mediator in metabolite signaling through the aryl hydrocarbon receptor (AhR). We tested if AhR is required for HCMV replication using CH223191, an AhR inhibitor that blocks KYN binding (20, 21). As we did for NLG919 treatment, we first examined the effect of CH223191 treatment on HCMV replication in fibroblast cells that had not been genetically modified. Cells were infected at an MOI of 1 and then treated with DMSO or CH223191 at concentrations ranging from 3 to 24 μM. The medium was replaced at 2 dpi to renew the level of CH223191. Under these conditions, a reduction of less than 5% cell survival was observed when uninfected cells were treated at 24 μM (Fig. S4A). Lower concentrations of CH223191 had little to no effect on cell survival. At 5 dpi, CH223191 treatment at 24 μM reduced HCMV replication by 11-fold (Fig. 5A). Treatment at 3 to 12 μM reduced HCMV replication by 2- to 7-fold. We further tested if HCMV replication depends on AhR activity by infecting NT and HIF1α KO cells at a low MOI. Again, we used the lowest concentration of inhibitor tested, and the medium was replaced every third day to renew the level of CH223191. At 9 days posttreatment, DMSO-treated uninfected cells and 3 μM CH223191-treated uninfected cells had the same level of cell survival (Fig. S4). At 9 dpi, HIF1α KO and NT cells treated with CH223191 produced fewer infectious progeny than those treated with DMSO (Fig. 5B).

Conversely, activation of AhR with an exogenous dioxin ligand enhances HCMV replication (22). We tested if AhR activation by an exogenous ligand would enhance HCMV replication by treating cells with the AhR activator 2,3,7,8-tetrachlorodibenzo-*p*-dioxin (TCDD). Since TCDD is a liquid at room temperature, we diluted TCDD directly in the cell growth medium. As a control, we added an equal volume of water to the growth medium. Since TCDD is known to promote HCMV replication in primary human fibroblasts at 3.1 nM, we examined if TCDD treatment at this level would alter HCMV replication in NT and HIF1α KO cells. At 9 days posttreatment, cells survived equally in water and 3.1 nM TCDD treatment (Fig. S4). In NT cells, HCMV infectious-progeny production was 2.5-fold higher in TCDD-treated cells than water-treated cells (Fig. 5C). In HIF1α KO cells, virus progeny production was 1.5-fold higher in TCDD-treated cells than water-treated cells (Fig. 5C).

### KYN enhances HCMV replication.

**FIG 6:**
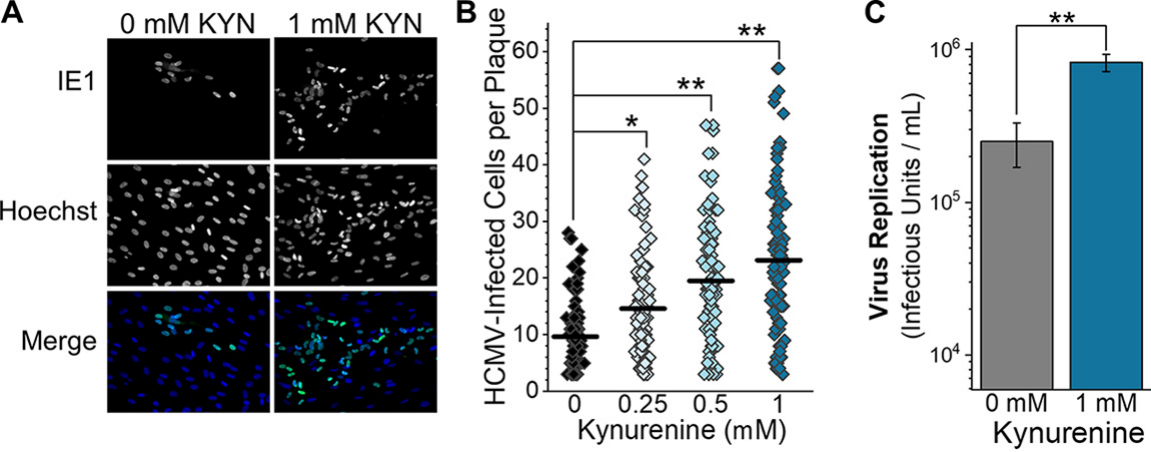
Feeding KYN to cells promotes HCMV infection. (A) HCMV replication and spread in fibroblast cells treated with KYN was measured. Cells were infected with 100 infectious units for 1 h and then washed with PBS. The infected cells were then overlaid with growth medium containing 0, 0.25, 0.5, and 1 mM KYN and 0.6% methylcellulose. At 3 dpi, the growth medium was replaced to replenish KYN. At 6 dpi, the cells were washed and fixed with methanol. The nuclei of infected cells were visualized using immunofluorescence and an antibody against HCMV IE1 protein. All nuclei were visualized by staining with Hoechst 33342. Representative images from cells treated with 0 and 1 mM KYN are shown. (B) The number of IE1-positive cells per plaque was determined in cells treated with KYN, as described for panel A. At least 10 plaques were counted per experiment. In order to be counted, a plaque had to contain at least three IE1-positive cells. The mean is represented as a black line, and significance was determined by Kruskal-Wallis ANOVA and Mann-Whitney test (*n* = 6) (*, *P* < 0.05; **, *P* < 0.01). (C) Infectious virus progeny produced by 0 mM and 1 mM KYN-treated HFFs was measured by TCID_50_ at 9 dpi. Cells were infected at an MOI of 0.05, washed, and then treated. The growth medium was replaced at 3 and 6 dpi to replenish KYN levels. The data are means ± SD (*n* = 3). Paired *t* test (**, *P* < 0.01).

Based on our observations, we hypothesize that KYN enhances HCMV replication. We tested this hypothesis by feeding KYN to cells following a low-MOI infection and monitoring HCMV spread in cell culture. In this experiment, we used primary human fibroblast cells that had not been genetically altered by CRISPR/Cas9 or any other means. At 1 hpi, we washed the cells and then fed the cells growth medium supplemented with 0, 0.25, 0.5, or 1.0 mM KYN. At 6 dpi, we fixed and visualized infected cells using an antibody against HCMV IE1 protein (Fig. 6A). Since IE1 is localized in the nucleus, we determined the number of IE1-positive nuclei per plaque. We counted the number of infected cells per plaque in 6 independent experiments for a total of ≥80 plaques for each KYN concentration tested. In untreated cells, the average plaque size was 10 cells per plaque. In KYN-treated cells, the average plaque size was 15 to 23 cells (Fig. 6B). Since plaque size allows us to visualize HCMV replication and cell-to-cell spread, we next examined if KYN treatment would affect HCMV replication by measuring infectious-progeny production at 9 dpi. In this case, fibroblast cells were infected at an MOI of 0.05 infectious unit per cell, washed, and fed growth medium containing 0 or 1 mM KYN. At 9 dpi, the amount of infectious progeny was measured by TCID_50_. KYN treatment increased the level of HCMV produced by 3-fold (Fig. 6C). Since KYN treatment increased HCMV plaque size at 6 dpi and infectious-progeny production at 9 dpi, we conclude that KYN promotes HCMV replication.

## DISCUSSION

Viral reprogramming of host metabolism is essential for HCMV replication (2, 23–25). HCMV infection reprograms the flow of nutrients through metabolic pathways by altering host metabolic regulators (5, 7, 8, 26). However, most studies have focused on how host metabolic regulation supports virus replication. Here, our findings show that the host uses HIF1α as a metabolic regulator to reduce virus replication.

McFarlane and colleagues demonstrated that UV-inactivated HCMV virus particles triggered HIF1α expression in cells in normoxia (9), consistent with the idea that the host activates HIF1α to contribute to an antiviral response. Our work extends this concept by demonstrating that IDO1 expression, KYN levels, and virus replication are suppressed via a HIF1α-dependent mechanism in HCMV-infected cells in the presence of oxygen. IDO1 is a gamma interferon (INFγ)-inducible gene. INFγ-induced IDO1 gene expression and activity are reduced by HCMV infection (27–31). Our findings suggest that this observed reduction of IDO1 activity by HCMV infection could be due to a HIF1α-dependent host response to suppress HCMV replication. Moreover, hypoxia represses the induction of IDO1 in uninfected cells (32, 33), providing further evidence supporting the role of HIF1α in regulating IDO1 activity.

Our findings suggest that HIF1α suppresses IDO1 expression and KYN synthesis to reduce HCMV replication. Based on our observations, we propose that the host cell uses a HIF1α-dependent response to reduce IDO1 activity and limit KYN levels to dampen AhR signaling and reduce HCMV replication. In the absence of HIF1α, HCMV infection enhances IDO1 synthesis of KYN. Subsequently, KYN metabolite-mediated activation of AhR signaling would promote HCMV replication. Intracellular and extracellular KYN levels are elevated in HIF1α KO cells, suggesting that KYN activation of AhR may occur within infected cells and neighboring uninfected cells. We found that TCDD activation of AhR promoted HCMV replication (Fig. 5B). According to our proposed model, TCDD activation of AhR would be lower in HIF1α KO cells, since those cells would have a higher level of AhR activation by KYN. Indeed, we observed that TCDD treatment promoted HCMV replication to a lesser extent in HIF1α KO cells than NT cells (Fig. 5B).

In this study, we found that HCMV infection raises the intracellular and extracellular concentration of KYN (Fig. 3A and B). Immunosuppressed HCMV-infected transplant recipients have elevated KYN serum levels (18). Additionally, people with HCMV and human immunodeficiency virus type 1 (HIV-1) coinfection have enhanced KYN serum levels relative to those with HIV and herpes simplex virus 1 (HSV-1) coinfection (19). While our observations demonstrate that HCMV infection increases KYN levels in cell culture, it remains unknown if elevated KYN serum levels *in vivo* are altered due to the metabolic activity of HCMV-infected cells or uninfected cells responding to infection. KYN functions as a metabolite in tryptophan catabolism and as a mediator in AhR signaling. Blocking AhR activity limits HCMV replication in HIF1α KO and control cells (Fig. 5A). Conversely, activation of AhR enhances HCMV replication (Fig. 5B) (22). While additional studies are needed, these observations suggest that elevated KYN levels in human serum may promote infection.

HIF1α suppressed HCMV replication at a low MOI but not at a high MOI (Fig. 1). A low-MOI infection measures multiple rounds of virus replication that allows cell-to-cell spread. One possible explanation for the suppression of virus replication by HIF1α at a low MOI is that HIF1α may reduce HCMV cell-to-cell spread, and by further extension, KYN may promote cell-to-cell spread. This possibility is consistent with KYN treatment enhancing HCMV plaque sizes (Fig. 6A and B). Another explanation for suppression of virus replication by HIF1α at a low MOI but not at a high MOI is that large amounts of HCMV particles may reduce the ability of HIF1α to reduce virus replication. In this context, HCMV may encode a mechanism that targets HIF1α to limit its activity in suppressing virus replication. In support of this possibility, we find that HIF1α protein levels are reduced at 3 and 4 dpi (Fig. 1A and B). Finally, our observation that HCMV replicates better in HIF1α KO cells in a low-MOI assay could be due to small replication differences that are amplified in the multicycle replication assay but not observable using a high-MOI single-cycle replication assay. These observations highlight the need for further research to understand the dynamics between HCMV and HIF1α.

We found that the levels of several metabolites are HIF1α dependent (Fig. 2B). The identification of KYN allowed us to define a mechanistic connection between HIF1α, IDO1, and AhR during HCMV infection. Several additional metabolites regulated by HIF1α remain unknown in our untargeted metabolomics data (Fig. 2). These include two peaks—peak 1 and 4—that were higher in both intracellular and extracellular environments of HIF1α KO cells than in those of NT cells (Fig. 2). These unidentified metabolites may also regulate HCMV replication, and their future identification may result in new understandings of the interaction of HCMV and host metabolism. Some of them, like KYN, may be reduced by HIF1α to limit HCMV replication.

In addition to metabolism, HIF1α regulates other cellular systems, including transcription, proliferation and survival, and immune responses (34, 35). While we found that HIF1α acts as a regulator of metabolism by suppressing KYN levels, the possibility remains that HIF1α may further affect HCMV infection through its other functions. More research that examines the possible effect of additional functions of HIF1α on virus replication is needed to define any additional HIF1α-dependent mechanisms cells may use to repress HCMV. Additionally, IDO1 and AhR have broad impacts on biology, including metabolism and immune regulation (36–38). Future studies focusing on the role of IDO1 and AhR in virus replication will likely reveal several new findings in HCMV biology.

Overall, we discovered a metabolic pathway regulated by HIF1α in HCMV infection in a way that suppressed virus replication. These findings suggest that HIF1α acts as an antiviral factor through its activity as a metabolic regulator. HIF1α and hypoxia have been linked to innate defenses against other viruses (39, 40), parasites (32, 41), and bacteria (42), suggesting that HIF1α regulation of metabolism may have broad impacts on microbial infections. Our work described here provides a foundation for future work to understand how HIF1α may modulate metabolism to support innate cellular antimicrobial defenses.

## MATERIALS AND METHODS

### Cells and virus infection and virus replication.

All experiments were performed under normoxic conditions using HCMV strain TB40/E or TB40/E-GFP (43, 44). Virus stocks were grown from fibroblasts electroporated with a bacterial artificial chromosome containing the TB40/E-GFP genome (BAC4-TB40/E-GFP), which was kindly provided by Thomas Shenk (Princeton University). Infectious HCMV was purified and concentrated from the supernatant of infected cells by ultracentrifugation through 20% sorbitol. Viruses were titrated using TCID_50_. Human foreskin fibroblasts (HFFs) were grown and maintained in Dulbecco’s modified Eagle medium (DMEM) containing 4.5 g/liter glucose (Gibco no. 11965-092), supplemented with 10% fetal bovine serum (FBS; Sigma-Aldrich no. 12303C), penicillin and streptomycin (Pen/Strep), and 5 mM HEPES (pH 7.4) (Gibco no. 15630-080).

One day prior to infection or mock infection (i.e., uninfected samples), 1.6 × 10^5^ cells per well were seeded on 6-well plates. Cells were either mock infected or HCMV infected for 1 to 2 h at the indicated multiplicity of infection (MOI). After mock or HCMV infection, cells were washed with phosphate-buffered saline (PBS) and provided DMEM or RPMI 1640 growth medium containing 10% FBS, HEPES, and Pen/Strep.

Virus replication assays were performed in RPMI 1640 (Gibco 11875-093) supplemented with 10% FBS, 5 mM HEPES, and Pen/Strep. RPMI 1640 was used for replication assays since this medium contains a concentration of tryptophan that is more similar to the level of tryptophan in serum than DMEM. Following infection, cells were washed with PBS, and 1.5 ml of RPMI 1640 growth medium was added to each well. Samples were collected at 0, 2, 5, 9, 12, or 16 days postinfection (dpi). Viruses were harvested by scraping the wells and collecting cells and media. Samples were stored at −80°C. After thawing, each sample was briefly sonicated to release cell-associated viruses. Finally, the volume of each sample was measured, and the amount of infectious virus per milliliter was determined using TCID_50_ assays. Reported viral titers were determined using 2 to 4 TCID_50_ plates per sample.

### Protein analysis by Western blotting.

SDS-PAGE was performed using Bio-Rad anyKD and 4 to 20% gradient gels. Proteins were transferred to nitrocellulose membranes (Li-Cor no. 926-31092), and blots were blocked using 3% milk in a Tris-buffered saline containing 0.05 to 0.1% Tween 20. The following antibodies were used to detect proteins: antitubulin (Sigma-Aldrich no. T6199), anti-IE1 (1B12) (45), anti-HIF1α (BD Biosciences no. 610959), anti-mouse DyLight 800 (Thermo Fisher no. SA5-35521), and anti-rabbit DyLight 680 (Thermo Fisher no. 35568). Anti-HIF1α antibody was incubated with blocked membranes at a 1:1,000 dilution for 14 to 16 h at 4°C. All other antibodies were incubated for 1 h at room temperature. Proteins were visualized and quantified using a Li-Cor Odyssey CLx imager and Image Studio software. Where noted, HIF1α protein levels were normalized using tubulin as a control. For experiments using cobalt chloride (CoCl_2_) to enhance HIF1α levels, cells were treated with a final concentration of 100 μM CoCl_2_ for 24 h prior to being lysed for Western blotting.

### Engineering of HIF1α-KO cells using CRISPR/Cas9.

Single guide RNA (sgRNA) sequences specific for the human gene HIF1α were designed using crispr.mit.edu and further selected for low off-target potential for both the human and HCMV genome using CasOFFinder (46). sgRNA sequences used in this study were CRISPR gRNA HIF1α c. 1 (5′-CCATCAGCTATTTGCGTGTG-3′) and CRISPR gRNA HIF1α c. 2 (5′-TGTGAGTTCGCATCTTGATA-3′). sgRNA were cloned into LentiCRISPR-v2, a gift from Feng Zhang (Addgene no. 52961) (47). For the engineering of nontargeting (NT) control cells, the control gRNA (5′-CGCTTCCGCGGCCCGTTCAA-3′) sequence from the human GeCKO v2 CRISPR knockout library was cloned into the LentiCRISPR-v2 plasmid (47, 48).

HFFs were used for generating HIF1α KO clones. First, human telomerase (hTERT) was exogenously expressed using pBABE-neo-hTERT (Addgene no. 1774; a gift from Bob Weinberg [49]). HFF-hTERT cells were transduced with LentiCRISPR-v2 lentiviruses that carried the gRNA and Cas9. Transduced HFF-hTERT cells were selected using puromycin (Corning no. 61-385-RA). Next, selected cells were diluted to one cell per well on a 96-well plate and supplemented with 150 nontransduced HFFs. Once cells reached 90% confluence in most wells, puromycin was added to selected against the nontransduced HFFs. Surviving cells were grown up, and potential HIF1α KO clones were initially identified by Western blotting for HIF1α expression following a 24-h treatment with CoCl_2_. The loss of HIF1α was further confirmed by sequencing using the Guide-it indel kit (TaKaRa no. 631444) and primers targeting the corresponding genomic regions. The primers used were Guide-it indel sequencing primer 1 (5′-CACCTGCTTCCGACAGGTTT-3′) and primer 2 (5′-GGAAACACCTGCTTCCGACA-3′). Each clone was sequenced at least 20 times to ensure biallelic mutations in the HIF1α gene.

### Engineering of HIF1α KO cells to re-express HIF1α protein.

HIF1α containing silent mutations in the Cas9 PAM recognition site was used to re-express HIF1α protein in HIF1α KO cells. The wild-type human HIF1α gene was obtained from plasmid pcDNA3-HIF1α (Addgene no. 18949; a gift from William Kaelin [50]). A G-to-A mutation at position 210 using site-directed mutagenesis removed the PAM recognition site for HIF1α KO clone 1 while maintaining the wild-type protein sequence of Arg at amino acid position 70. Next, the HIF1α PAM-mutant gene was inserted into pLVX-EF1a using a TaKaRa In-Fusion HD Cloning Plus kit. Finally, the HIF1α PAM-mutant gene was transferred into pLV-TRE-blasticidin from VectorBuilder using BamHI and XbaI to generate a plasmid termed pLV-TRE-HIF1α_G210A-blast. pLV-TRE is a lentivirus system for doxycycline-inducible expression of proteins in mammalian cells. pLV-TRE-HIF1α_G210A-blast was sequenced to confirm the PAM site mutation and to confirm that the gene would encode wild-type HIF1α protein. For the engineering of HIF1α KO cells that expressed either the HIF1α PAM mutant or GFP as a control, HIF1α KO clone 1 cells were first treated with lentivirus particles from 293T cells transfected with pLV-rtTA-hygro. Next, HIF1α KO-rtTA cells were treated with lentivirus particles generated in 293T cells transfected with pLV-TRE-HIF1α_G210A-blast or pLV-TRE-GFP_blast. All pseudolentiviruses were produced using pMD.2G and psPAX2, as previously described (5). Doxycycline was used to induce the expression of HIF1α or GFP in HIF1α KO clone 1 cells. For KYN measurements in HIF1α KO cells expressing HIF1α or GFP, the cells were HCMV infected for 1 h, washed, and then provided fresh growth medium containing 1 μg doxycycline. GFP expression was confirmed using a fluorescence microscope, and HIF1α expression was measured by Western blotting.

### Metabolite extraction.

For all metabolomic experiments, duplicate samples were extracted in parallel for each condition. For extraction of extracellular metabolites, 150 μl of growth medium was harvested from plates containing either mock-infected or HCMV-infected cells. The extracellular growth medium was centrifuged at 21,000 × *g* for 5 min to pellet cell debris. Next, 100 μl of the resulting supernatant was used for metabolite extraction. Metabolites were extracted by adding cold methanol for a final concentration of 80% methanol. Subsequently, the samples were incubated on dry ice or at −80°C for 10 to 15 min and centrifuged at 4,000 × *g* at 4°C to remove cell debris and proteins. Finally, the extracted metabolites were dried under nitrogen gas.

Intracellular metabolites were methanol extracted as previously described (3, 26). First, the growth medium was removed, and cells were quickly washed with PBS. Next, cold 80% methanol was added to quench all metabolic reactions. Metabolites were extracted following incubation on dry ice or at −80°C for 10 to 15 min and centrifuged at 4,000 × *g* at 4°C. Extracted metabolites were dried under nitrogen.

We identified possible contaminants using controls that lacked cells. In this case, the growth medium was placed in a 6-well plate that lacked cells and analyzed in parallel to samples from 6-well plates that contained cells. The LC-MS/MS signal from the “no cell” samples was used to define the background to remove contaminants from our data sets.

### Metabolomics.

All metabolites were identified and quantified using liquid chromatography-tandem mass spectrometry (LC-MS/MS). Extracellular metabolites were normalized by extraction volume, and intracellular metabolites were normalized by cell volume (17, 26, 51). Metabolites were analyzed by reverse-phase chromatography or hydrophilic interaction liquid chromatography (HILIC). For reverse-phase analysis, metabolites were resuspended in MS-grade water. For HILIC analysis, metabolites were resuspended in 1:1 MS-grade water and MS-grade methanol. All samples were analyzed within 24 h of extraction to limit metabolite degradation. Reverse-phase analysis was performed using a Kinetex 1.7-μm F5 ultraperformance LC (UPLC) column (Phenomenex no. 00F-4722-AN), while HILIC analysis used an Acquity BEH HILIC 1.7-μm column (Waters no. 186003462). All LC was performed using a Vanquish LC system (Thermo Fisher Scientific) using an autosampler that stored samples at 4°C and a temperature-controlled column holder that kept columns at 25°C. Two buffers were used for LC, 97:3 water-acetonitrile plus 0.1% formic acid (buffer A) and 100% acetonitrile (buffer B). Each LC run was 30 min using the following conditions: 0% B for 2 min, 10% for 3 min, hold at 10% B for 1 min, 20% B for 4 min, hold at 20% B for 1 min, 55% B for 7 min, hold at 55% B for 1 min, 96% B for 4 min, and hold at 96% B for 1.5 min (reverse-phase chromatography) and 95% B for 2.5 min, 40% B for 8 min, hold at 40% B for 7.5 min, and 95% B for 2.5 min (HILIC). All LC was performed at 0.25 ml/min, and the column was equilibrated between samples.

Untargeted metabolomics was performed using a Q-Exactive Plus orbitrap mass spectrometer (Thermo Fisher Scientific). MS1 data were collected by full scans from 60 to 900 *m/z* using a mass resolution setting of 140,000. Data-dependent MS/MS was performed using a TopN setting of 5. Additional settings used were 1e^6^ AGC and a 250-ms maximum injection time. Normalized collision energy was set at 40 or 45. MS2 data were collected using a resolution setting of 35,000 with 1e^5^ AGC, a 120-ms maximum injection time, and a 10-s dynamic exclusion. Metabolites were ionized using a heated electrospray ionization probe with the following settings: for negative mode, sheath gas flow, 20 arbitrary units (a.u.); auxiliary gas flow, 10 a.u.; sweep gas flow, 1 a.u.; spray voltage, 2.5 V; capillary temperature, 243°C; S-lens radio frequency (RF), 65 V; and auxiliary gas temperature, 205°C; for positive mode, sheath gas flow, 32 a.u.; auxiliary gas flow, 10 a.u.; sweep gas flow, 1 a.u.; spray voltage, 3.0 V; capillary temperature, 253°C; S-lens RF, 58 V; and auxiliary gas temperature 180°C. Metabolites were analyzed using MAVEN (52, 53). UPLC- or MS-grade solvents were used for LC-MS/MS analysis, including Optima water (Fisher no. W7-4), Optima LC/MS methanol (Fisher no. A456), Optima LC/MS acetonitrile (Fisher no. A955), and Optima LC/MS-grade formic acid (Fisher no. A117).

For untargeted analysis, peaks were selected using the following settings in MAVEN: mass resolution, 5 ppm; time resolution, 10 scans; minimum signal-to-baseline ratio, 5; minimum signal-to-blank ratio, 5; scan minimum peak width, 3; and minimum peak intensity, 2,000 ions. Background signal from samples that contained no cells was used to remove peaks from contaminants. Peaks from multiple replicates were combined using hierarchical clustering (Ward’s method) of *m/z* and retention time. Peaks had to have been present in three of six independent experiments to be included in further analysis. Peaks were further analyzed, as outlined in Fig. 2A. Briefly, peaks were retained for analysis if the total ion count was ≥1,000 ions and if they had a ≥10:1 signal-to-blank ratio. Additionally, peaks with a background signal of ≥10,000 ions were removed. Next, peaks with collected MS2 fragments were retained, while those without MS2 fragments were removed. MS/MS identification involved integration of MS2 data using a Python script (https://github.com/lisawise/maven_explorer). Finally, statistical analysis was performed on the remaining peaks, as described below.

Metabolite identification was based on three parameters: the retention time on the LC column, MS1 spectral information using a ±5 ppm mass accuracy range, and characteristic MS2 fragments. A library of retention time, MS1, and MS2 information under our LC-MS/MS conditions was generated using purified compounds from commercial sources, including the Metlin-tested mass spectrometry metabolite library (MSMLS) from Sigma-Aldrich.

Each metabolite from the MSMLS (Sigma-Aldrich) listed in Fig. S2 was directly infused into the mass spectrometer, and the optimum conditions for ESI and MS/MS fragmentation were defined. Metabolites were used to determine the ionization and MS settings used in the untargeted metabolomics studies. Further, these metabolite standards were used to build a MS2 fragment library used for metabolite identification. Kynurenine standard (Sigma-Aldrich K8625) was used to generate the data listed in Fig. 2C.

### Small-molecule inhibitor and activator treatment.

NLG919 (Cayman Chemicals no. 21509) was used to inhibit IDO1 activity. CH223191 (Tocris Bioscience no. 3858) was used to inhibit AhR, and 2,3,7,8-tetrachlorodibenzo-*p*-dioxin (TCDD; Cambridge Isotopes Laboratories no. ED-901) was used to activate AhR. NLG919 and CH223191 were suspended in DMSO (Sigma-Aldrich no. D2650). Cells treated with DMSO were used as a control for NLG919 and CH223191. Since TCDD is a neat compound (i.e., liquid at room temperature), molecular biology grade nuclease-free sterile water (Grow Cells NUPW-0500) was used as a control. For all experiments using small molecules, the compound was added to cells after infection. At 2 hpi, cells were washed with PBS. Cells were then fed growth medium that contained either NLG919, CH223191, TCDD, DMSO, or water. At 3 and 6 dpi, the growth medium was removed and replaced with medium containing fresh compounds. Cell viability under small-molecule treatment conditions was determined using a lactate dehydrogenase (LDH) release cytotoxicity assay kit (Thermo Pierce no. 88953).

### RT-qPCR.

Reverse transcription-quantitative PCR (RT-qPCR) was performed to determine the level of mRNA expressed in NT and HIF1α KO cells. RNA was isolated and purified from cells using a ZR-Duet DNA/RNA miniprep kit (Zymo Research). Next, 1 μg of RNA was converted to cDNA using an oligo(dT) primer and the Transcriptor first-strand cDNA synthesis kit (Roche Molecular Systems). qPCR was performed using PowerUp SYBR green (Applied Biosystems Thermo Fisher Scientific) on an ABI real-time qPCR 7300 instrument. H6PD was used to normalize all samples. The following primer pairs were used: IDO1 pair 1 (5′-GGCTTTGCTCTGCCAAATCC-3′ and 5′-TTCTCAACTCTTTCTCGAAGCTG-3′), IDO1 pair 2 (5′-GCATTTTTCAGTGTTCTTCGCATA-3′ and 5′-CATACACCAGACCGTCTGATAGCT-3′), VEGF (5′-TCCTCACACCATTGAAACCA-3′ and 5′-GATCCTGCCCTGTCTCTCTG-3′), and H6PD (5′-GGACCATTACTTAGGCAAGCA and 5′-CACGGTCTCTTTCATGATGATCT-3′).

### KYN treatment and visualization of HCMV plaques.

KYN (Sigma-Aldrich K8625) was suspended in 0.1 N HCl. A 1 mM concentration of KYN was added to the growth medium (RPMI 1640, 10% FBS, 5 mM HEPES [pH 7.4], Pen/Strep), and the pH was adjusted to 7.4 using 0.2 N NaOH. As a control, the same volume of 0.1 N HCl that lacked KYN and 0.2 N NaOH was added to the same volume of growth medium to generate the 0 mM KYN control growth medium. The 1 mM KYN growth medium was serially diluted with the 0 mM KYN growth medium to generate 0.5 mM and 0.25 mM KYN growth medium stocks. All growth medium was sterilized by filtration. HFFs were seeded onto a 6-well plate at a concentration of 1.6 × 10^5^ cells per well. Cells were infected with 100 infectious units per well with TB40/E for 1 h. After infection, the cells were washed with PBS, and 2 ml of growth medium containing 0, 0.25, 0.5, or 1 mM KYN and 0.6% methylcellulose was added. At 3 dpi, the growth medium was removed and replaced with fresh growth medium to replenish the levels of KYN. At 6 dpi, the cells were fixed with methanol. The IE1-positive nuclei were visualized using immunofluorescence using an antibody against IE1, as previously described (5). The IE1-positive nuclei were counted in at least 10 plaques per well, with a plaque being defined as having three or more IE1-positive nuclei. All nuclei were visualized using Hoechst 33342.

### Quantification and statistical analyses.

Unless otherwise specified, data are displayed as means and standard deviations (SD), and *n* equals the number of independent biological experiments. For untargeted metabolomics, statistical analysis was performed in consultation with the Statistical Consulting Lab at the BIO5 Institute, University of Arizona. The untargeted LC-MS/MS data used in this study formed 4 distinct data sets: (i) intracellular metabolites/positive ion acquisition, (ii) intracellular metabolites/negative ion acquisition, (iii) extracellular metabolites/positive ion acquisition, and (iv) extracellular metabolites/negative ion acquisition. Each of these four data sets was analyzed separately by importing them into R and log-transformed to approximate normality. Only features present in both HCMV-infected NT cells and HCMV-infected HIF1α KO cells were analyzed, so that imputation of missing data points between NT and HIF1α KO conditions was unnecessary. Linear mixed-effect models were used to model the log-transformed measurements, with biological and technical replicates treated as random effects. The model outputs for each feature were fold change (HCMV-infected HIF1α KO cells relative to HCMV-infected NT cells) and *P* value, which were adjusted for multiple comparisons using the Benjamini-Hochberg procedure (54). These analyses were used to generate the volcano plots in Fig. 2B. OriginPro 2019 was used for all other statistical analyses.
