# Supplementary figures and images for: Hypoxia-Inducible Factor 1α (HIF1α) Suppresses Virus Replication in Human Cytomegalovirus Infection by Limiting Kynurenine Synthesis
Source: mBio. 2021 Mar 23;12(2):e02956-20. doi: 10.1128/mBio.02956-20 (PMC8092273; doi:10.1128/mBio.02956-20)

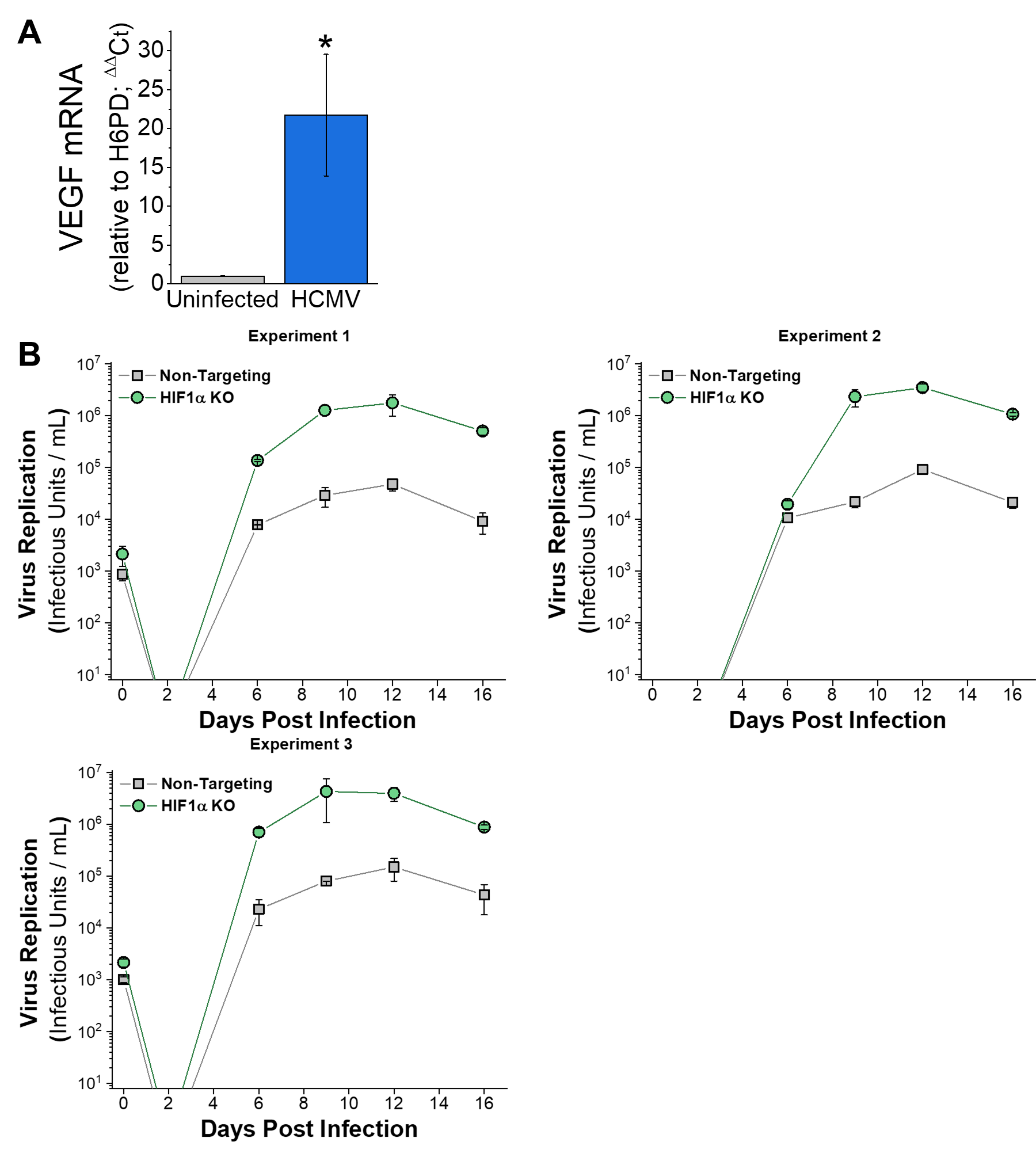

Supplement: FIG S1 [file mBio.02956-20-sf001.tif]

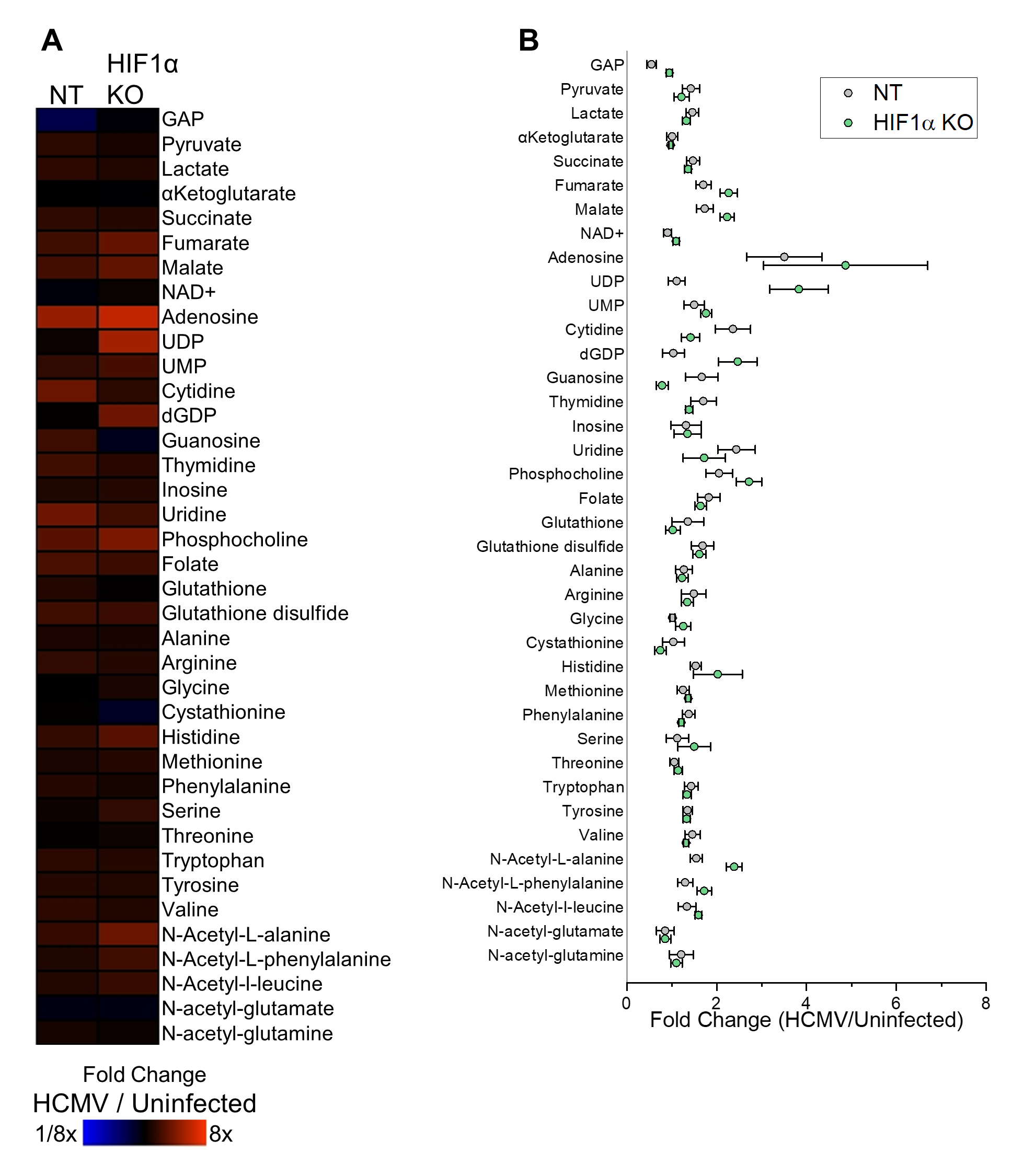

Supplement: FIG S2 [file mBio.02956-20-sf002.tif]

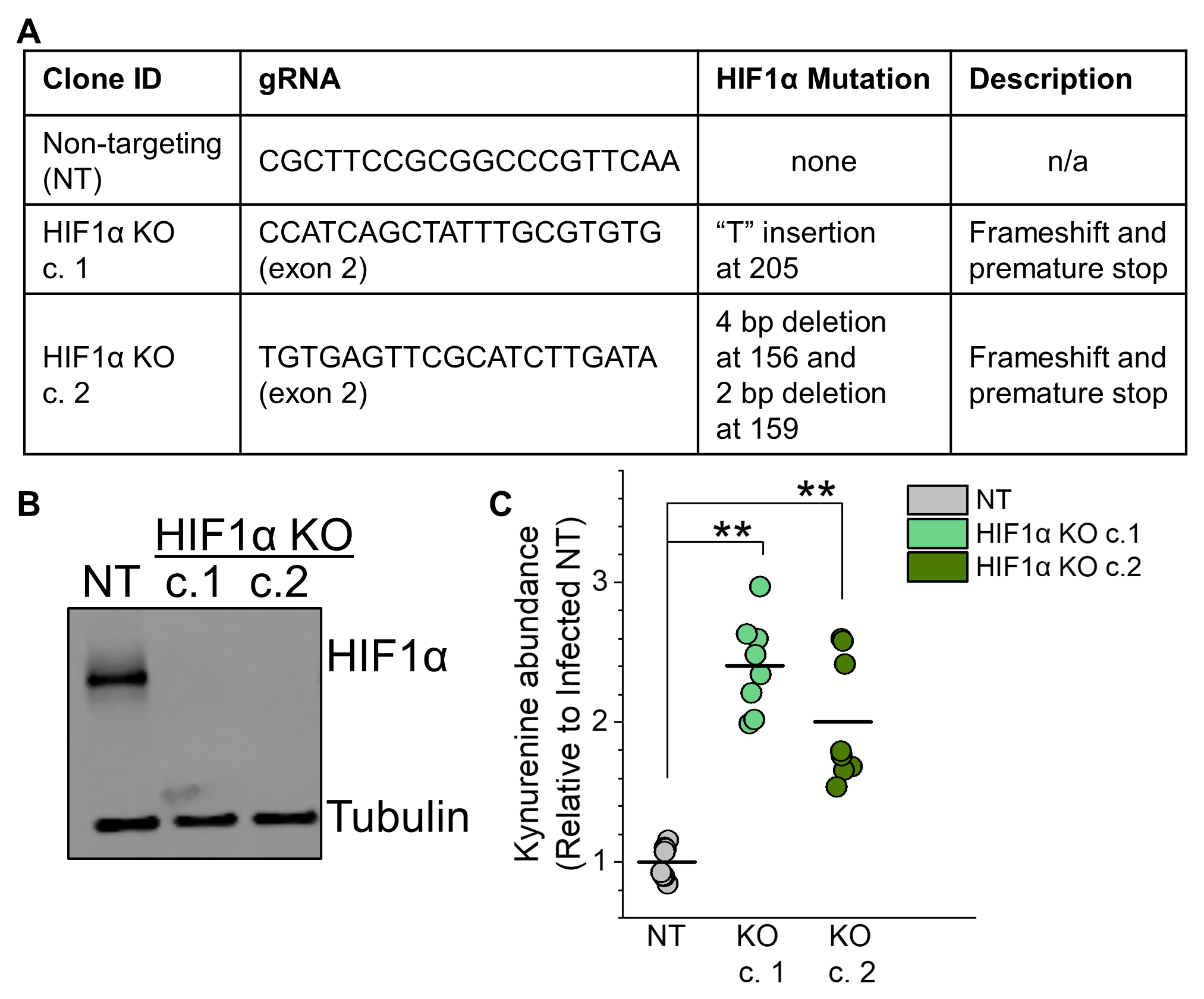

Supplement: FIG S3 [file mBio.02956-20-sf003.tif]

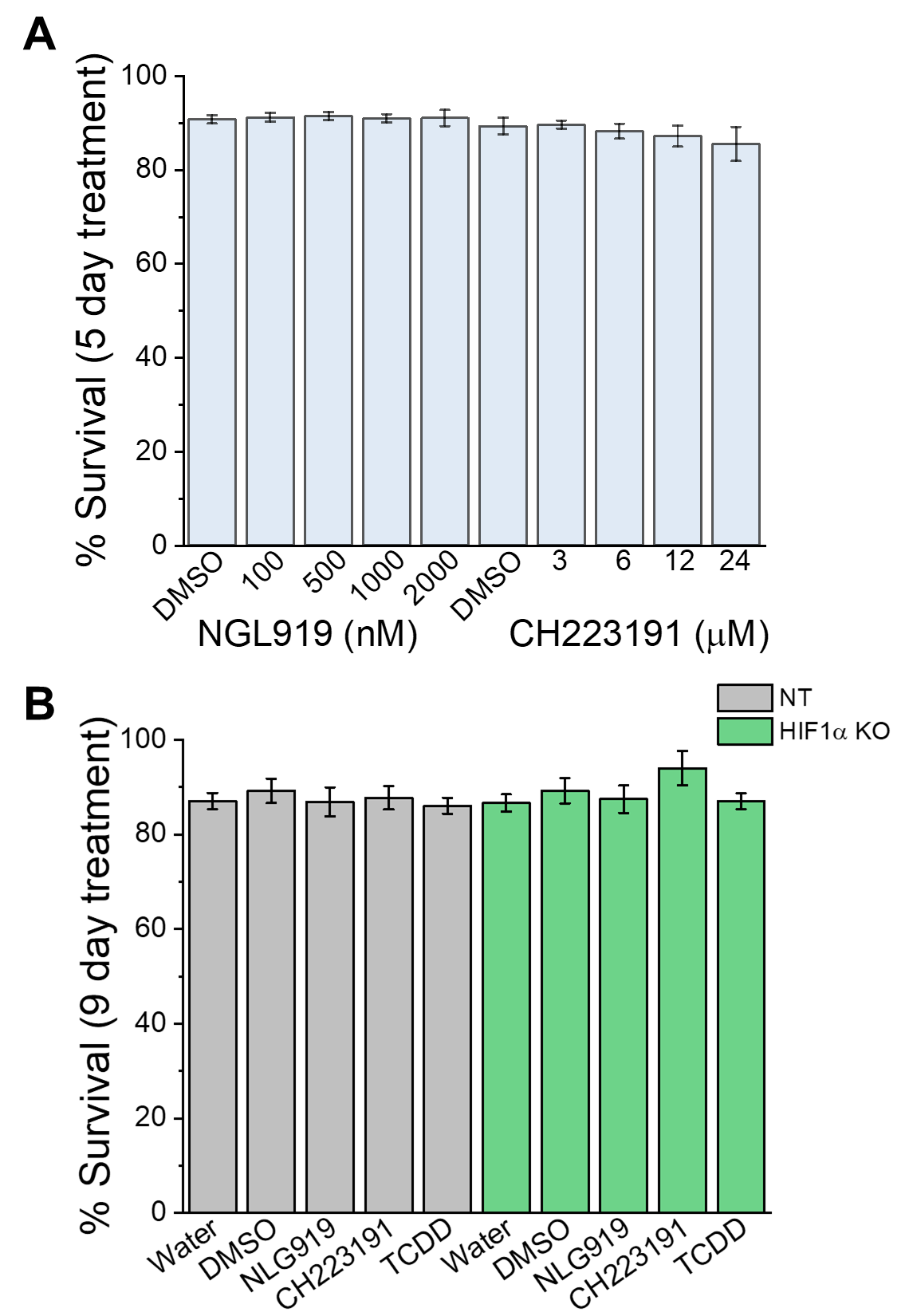

Supplement: FIG S4 [file mBio.02956-20-sf004.tif]
